# Complete Genome Sequence of *Lactobacillus plantarum* Strain 16, a Broad-Spectrum Antifungal-Producing Lactic Acid Bacterium

**DOI:** 10.1128/genomeA.00533-13

**Published:** 2013-08-01

**Authors:** Sarah Crowley, Francesca Bottacini, Jennifer Mahony, Douwe van Sinderen

**Affiliations:** Department of Microbiology, University College Cork, Cork, Irelanda; Alimentary Pharmabiotic Centre, University College Cork, Cork, Irelandb

## Abstract

*Lactobacillus plantarum* strain 16 restricts the growth of various food spoilage fungi and has the potential to be used as a biopreservative to improve the shelf life of foods. The complete genome sequence contains 3,044,678 bp with a G+C content of 44.74% and harbors the largest plasmid complement reported for this species to date.

## GENOME ANNOUNCEMENT

Lactic acid bacteria (LAB) have a long history of use in a diversity of food fermentations, where they contribute to the organoleptic properties and safety of the final product (1). The development of fungicidal resistance together with consumer trends toward safer methods of food preservation has fueled interest in the use of antifungal LAB as biopreservatives (2, 3). Members of the *Lactobacillus* genus, in particular strains of *Lactobacillus plantarum*, have become prominent players in the field of antifungal research (4, 5). 

*Lactobacillus plantarum* strain 16 (deposited as NCIMB41875) was originally isolated from malt production steep water and was determined to possess broad-spectrum antifungal activities. This strain was also shown to prevent the growth of certain fungal spoilers in several food models, including fruits, yogurt, and orange juice (6, 7).

Sequencing of *L. plantarum* 16 was performed by Macrogen (Seoul, Republic of Korea). A >120-fold sequencing coverage was obtained using pyrosequencing technology on a 454 FLX titanium instrument (using 3-kb mate-pair libraries). The files generated by the 454 FLX instrument were assembled with Newbler assembler v 2.0 (454 Life Sciences, Branford, CT) to generate a consensus sequence. Illumina sequencing using a Hiseq2000 platform was also performed, and the resulting 100-bp paired-end reads were assembled using Abyss v.1.3.4 (http://www.bcgsc.ca/platform/bioinfo/software/abyss) with an optimal k-mer size of 64. The Illumina data were subsequently mapped on the 454 consensus contigs to provide confidence in the sequence data and to provide accuracy in regions of homonucleotide polymeric tracts. The remaining gaps between scaffolds were closed by Sanger sequencing (MWG, Germany) of PCR-generated amplicons relating to these regions. Protein-encoding open reading frames (ORFs) were predicted using Prodigal v.2.60 (http://prodigal.ornl.gov). The ORF annotation was performed on the basis of BLASTP (8) analysis against the nonredundant protein database (nr) provided by the National Center for Biotechnology Information (NCBI). Artemis (9) was employed to inspect the results of the ORF prediction and its associated BLASTP results, which were used for a manual editing effort. Manual corrections to automated functional assignments were completed on an individual gene-by-gene basis as needed.

The complete genome sequence of *L. plantarum* 16 is composed of a single circular chromosome (3,044,678 bp) with an overall G+C content of 44.74%. The entire genome is predicted to contain 2,787 protein-coding genes, five rRNA operons, and 54 tRNA genes. It contains 8 completed plasmids, Lp16A (7,240 bp), Lp16B (8,636 bp), Lp16C (27,282 bp), Lp16D (37,097 bp), Lp16E (40,147 bp), Lp16F (50,195 bp), Lp16G (51,857 bp), and Lp16H (74,078 bp). Two additional scaffolds, Lp16I and Lp16L (13,341 and 6,464 bp, respectively), represent further plasmid-borne sequences but could not be closed, due to the presence of transposon-encoded regions. The sequenced genome represents one of the smallest of this species, while harboring the largest plasmid complement. Furthermore, the *L. plantarum* 16 genome contains 2 apparently complete prophage sequences and 51 annotated transposase-encoding genes.

### Nucleotide sequence accession numbers. 

The complete chromosome and plasmid sequences of *L. plantarum* 16 are deposited in GenBank under the accession numbers CP006033 (chromosome), CP006034 (Lp16A), CP006035 (Lp16B), CP006036 (Lp16C), CP006037 (Lp16D), CP006038 (Lp16E), CP006039 (Lp16F), CP006040 (Lp16G), CP006041 (Lp16H), CP006042 (Lp16I), and CP006043 (Lp16L).
